# Supplementary figures and images for: Intracortical neural activity distal to seizure-onset-areas predicts human focal seizures
Source: PLoS One. 2019 Jul 22;14(7):e0211847. doi: 10.1371/journal.pone.0211847 (PMC6645464; doi:10.1371/journal.pone.0211847)

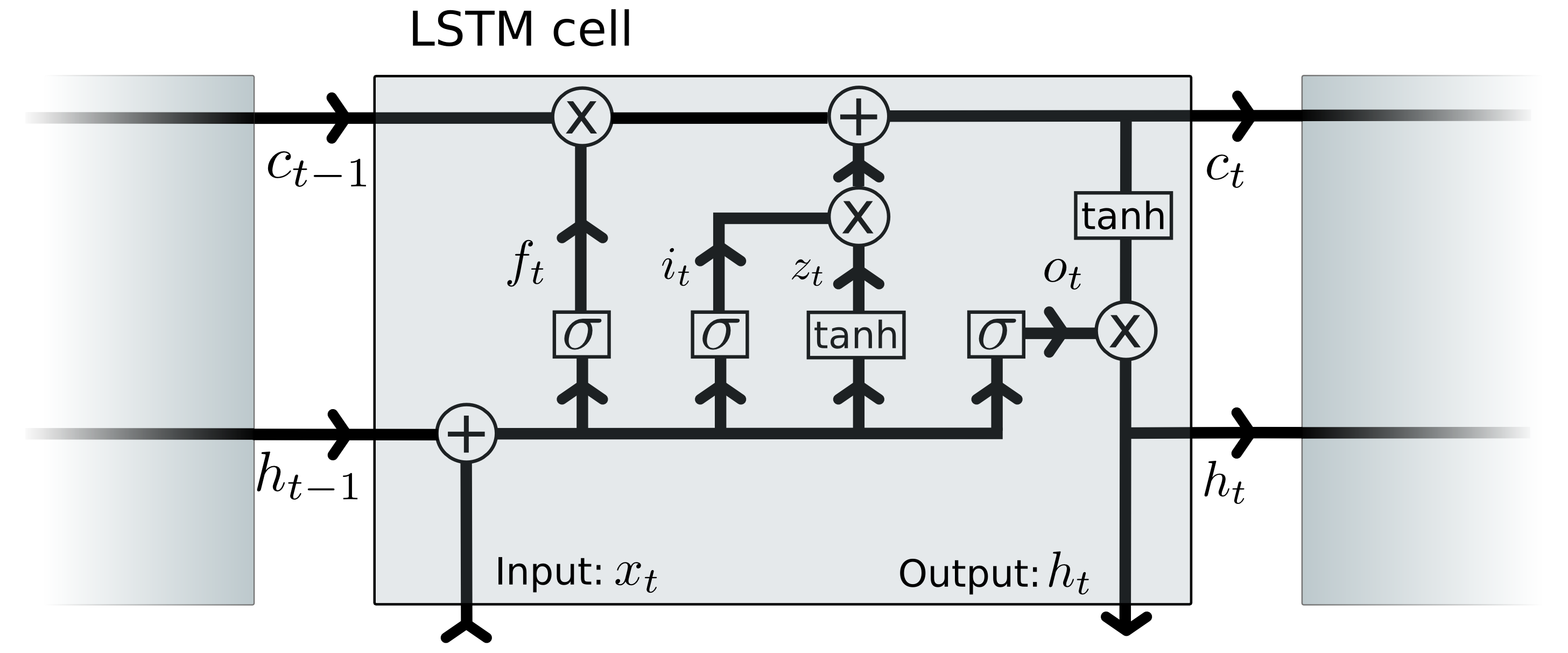

Supplement: S1 Fig — The state of the cell at time t, c(t), is updated at each time step with the former output of the cell, h(t-1), and new inputs, x(t), depending of the value of the input gate, i(t). The memory of the cell is controlled by the forget gate, f(t), while the output h(t) is controlled by the output gate, o(t). (TIF) [file pone.0211847.s001.tif]

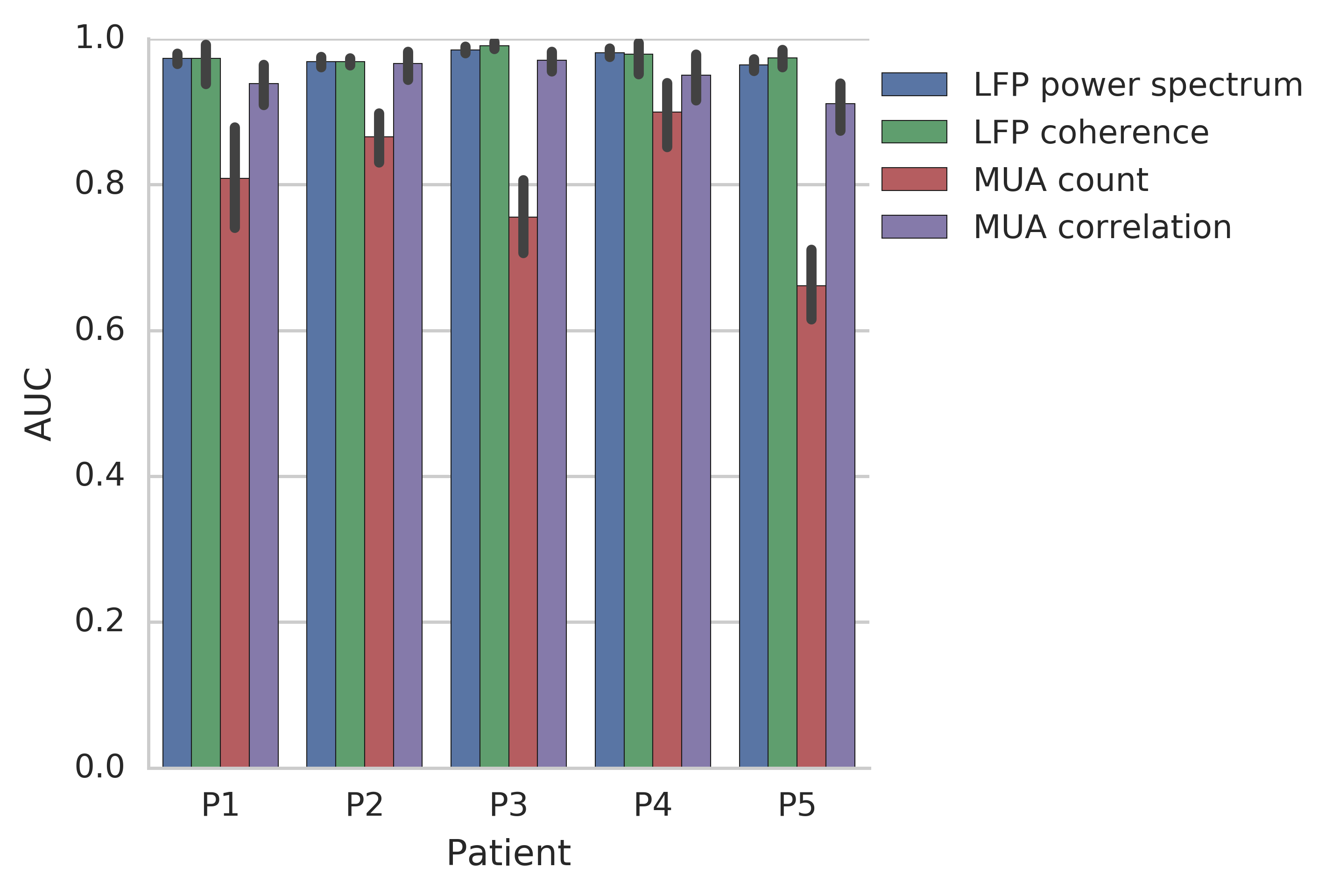

Supplement: S2 Fig — The bars indicate the AUC score averaged over all seizures for each patient and feature group according to an alternative cross-validation scheme where the order of the time windows are randomly permutated before assignment to training and test datasets. Blue: LFP power spectrum; Green: LFP pairwise spectral coherence matrix; Red: MUA count; Purple: MUA pairwise correlation matrix. (TIF) [file pone.0211847.s002.tif]

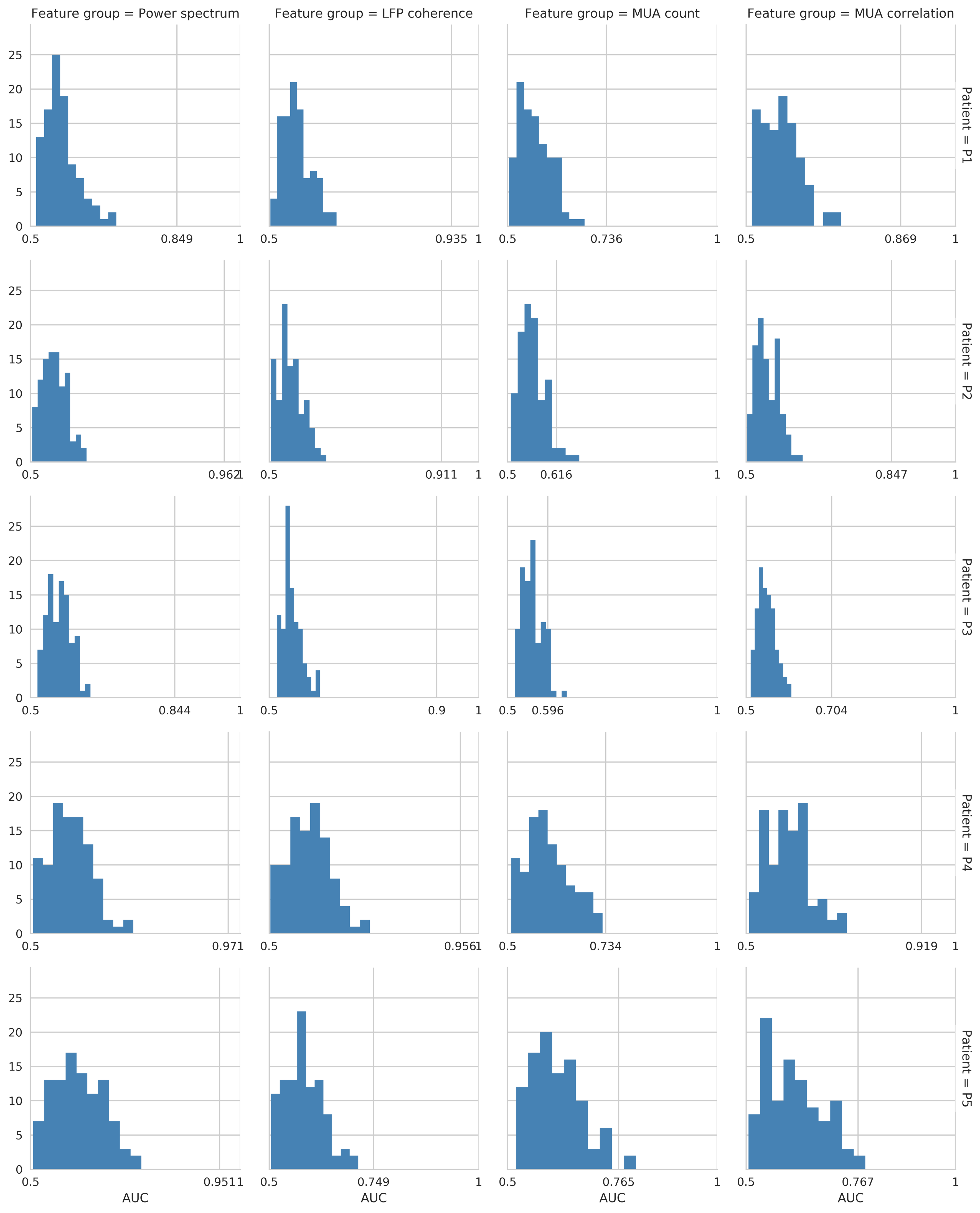

Supplement: S3 Fig — Each row corresponds to a different patient, and each column to a different group of features. The vertical line indicates the AUC score of the classifier when computed on the true (not chance-level surrogate) dataset. (See main text, Materials and methods, for details). (TIF) [file pone.0211847.s003.tif]

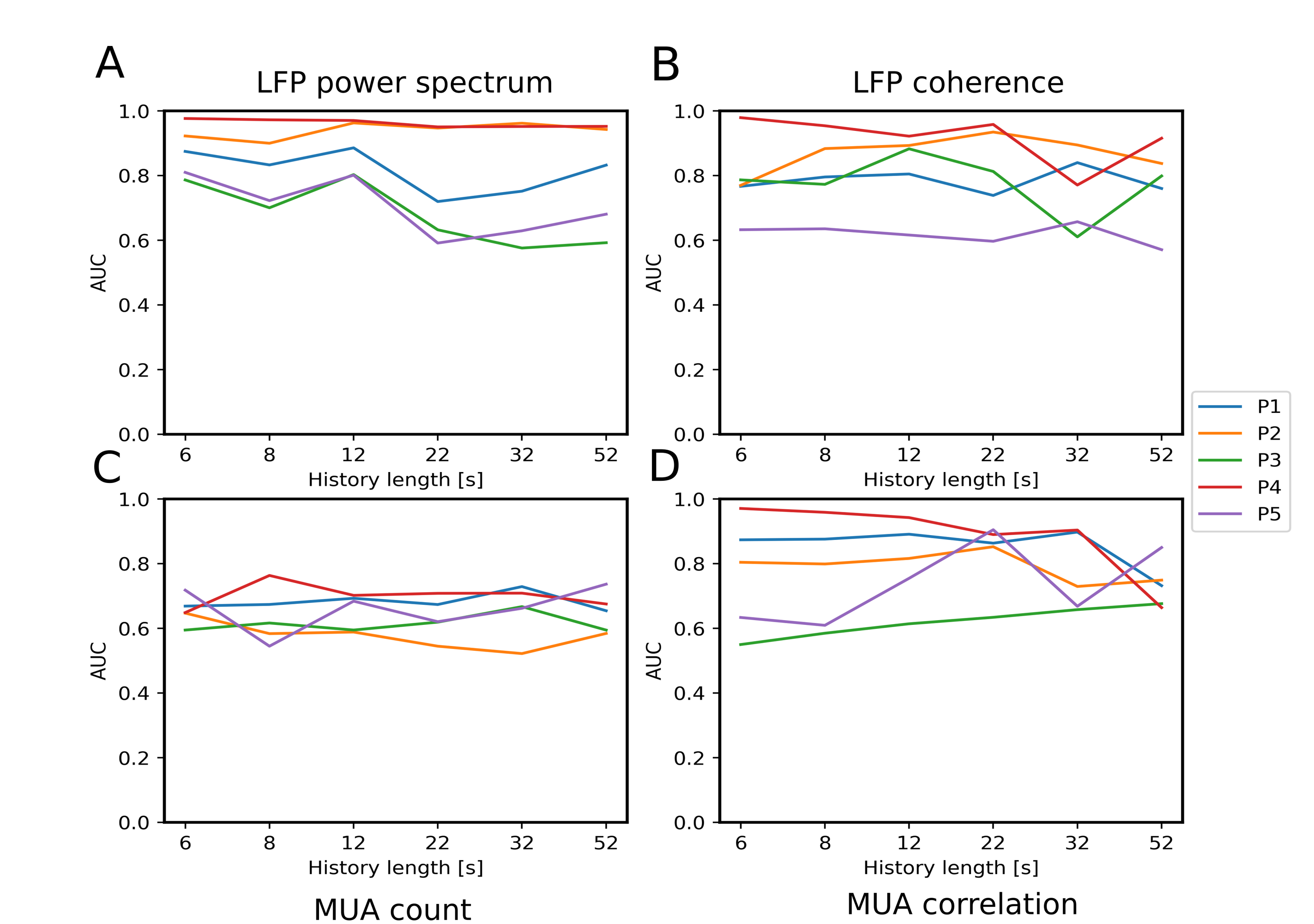

Supplement: S4 Fig — (A) LFP power spectrum (B) LFP coherence (C) MUA count (D) MUA correlation. Each curve corresponds to the AUC average computed across the test datasets for each patient. (TIF) [file pone.0211847.s004.tif]

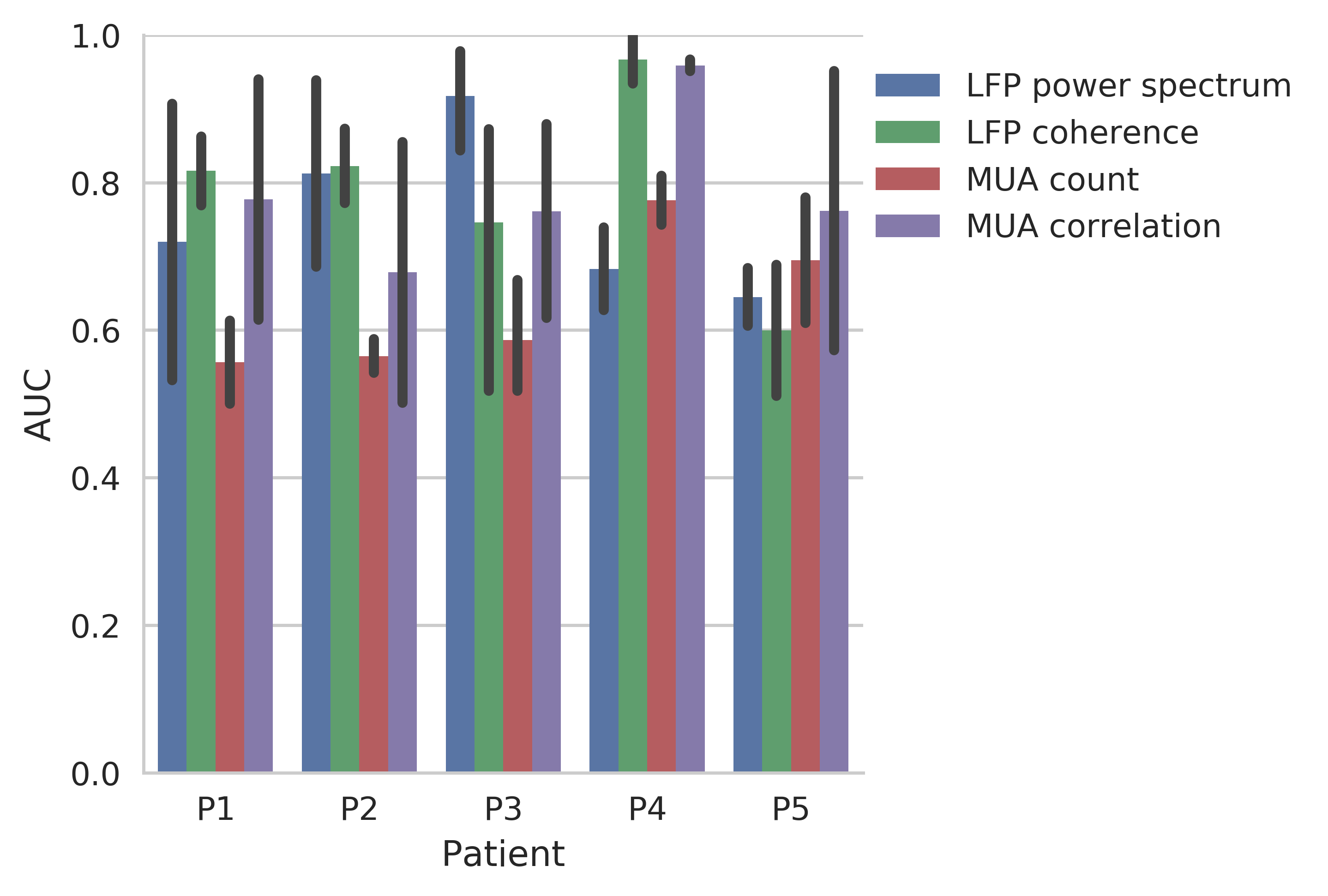

Supplement: S5 Fig — The bars indicate the AUC scores averaged over all seizures tested in the leave-one-out cross-validation setting for each patient and each feature. Blue: LFP power spectrum; Green: LFP pairwise spectral coherence matrix; Red: MUA count; Purple: MUA pairwise correlation matrix. (TIF) [file pone.0211847.s005.tif]

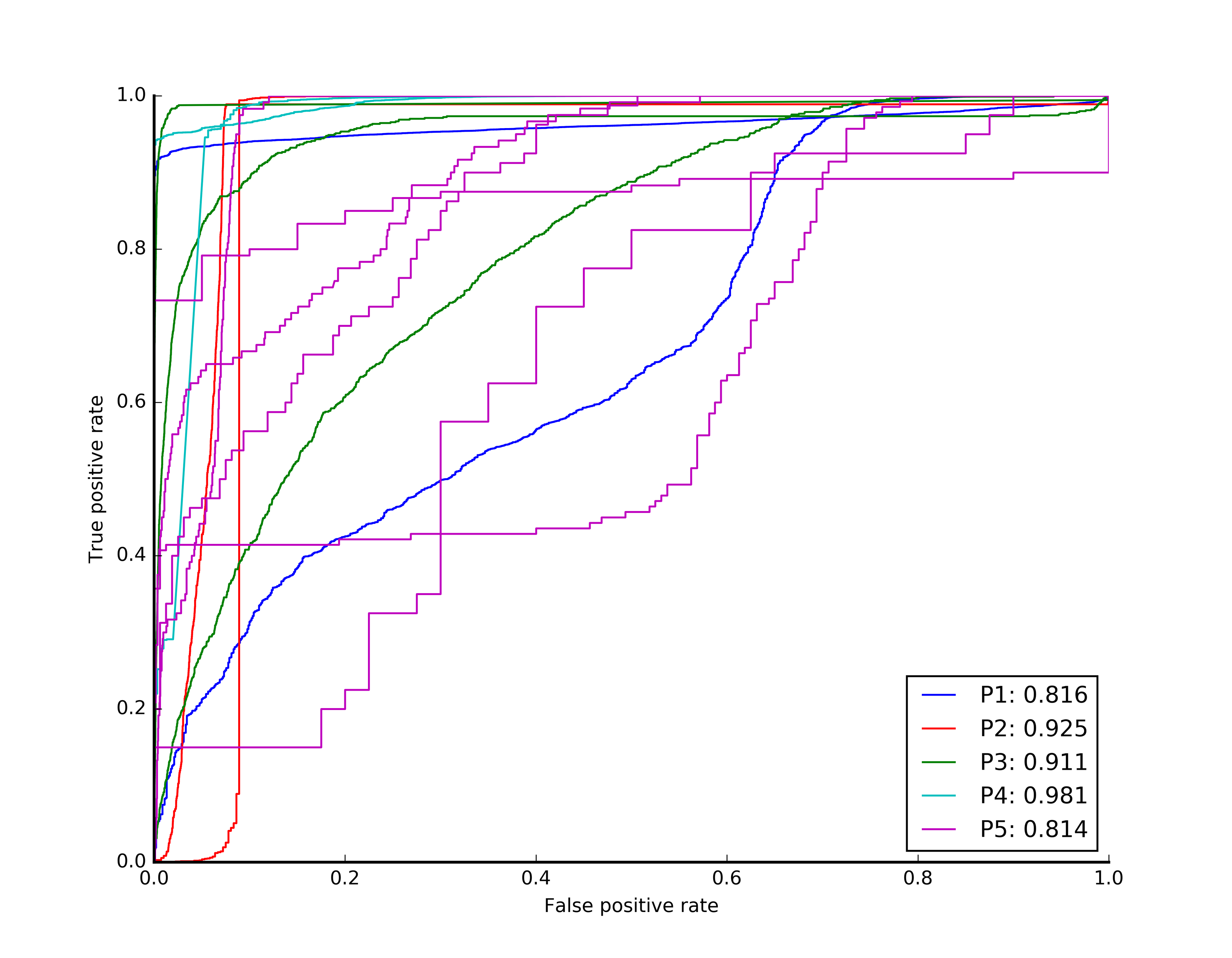

Supplement: S6 Fig — The legend indicates the corresponding prediction based on the average AUC score. (TIF) [file pone.0211847.s006.tif]

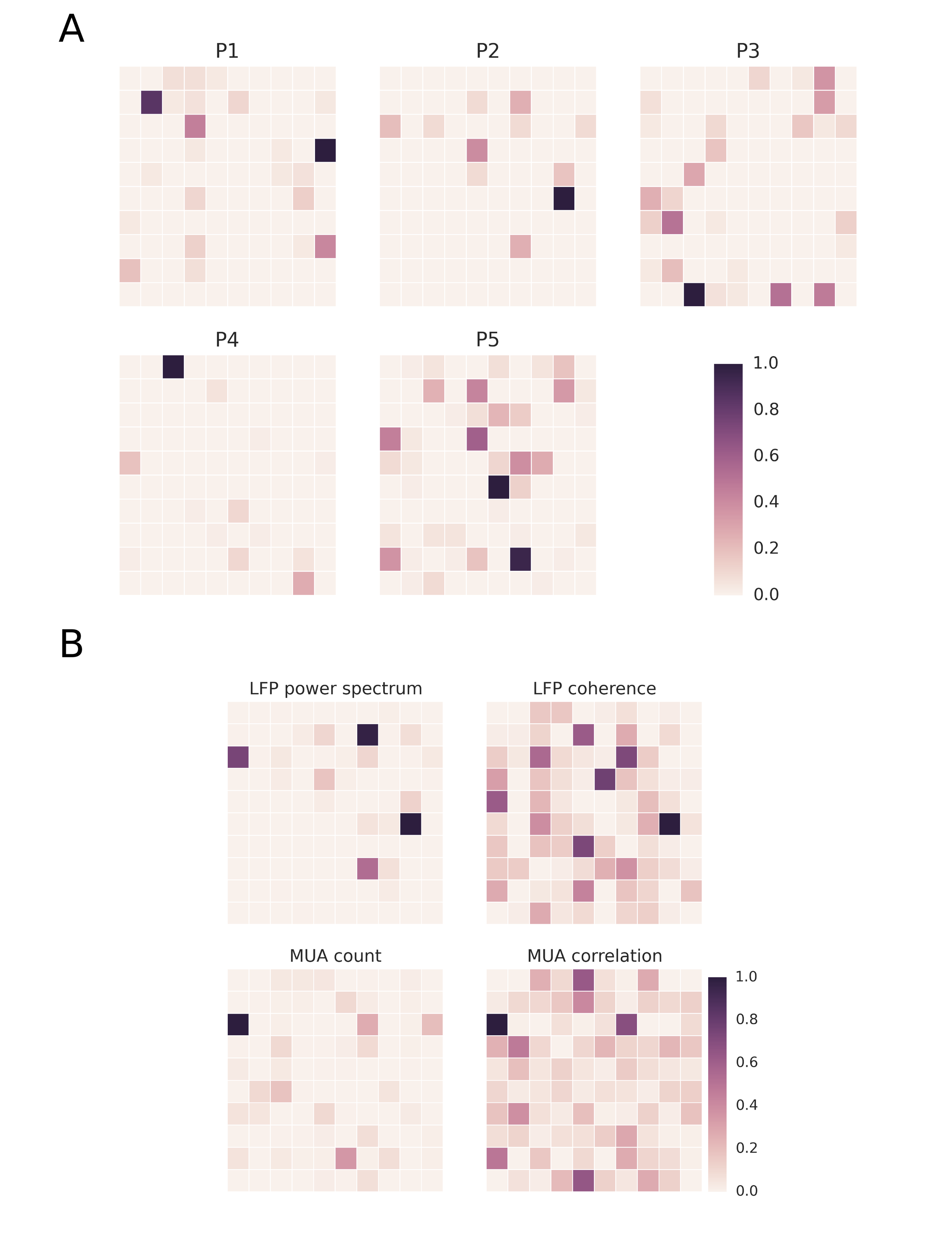

Supplement: S7 Fig — (A) MUA count feature. (B) Patient P3. LFP power spectrum, frequency band 300–500 Hz. The importance of a given MEA site is ranked from 0 to 1. MEA sites contributing to high importance features can be sparse and very localized in the 4 X 4 mm2 neocortical patch, indicating the existence of predictive microdomains of neural activity. (TIF) [file pone.0211847.s007.tif]
